# Antimicrobial Peptides: A New Hope in Biomedical and Pharmaceutical Fields

**DOI:** 10.3389/fcimb.2021.668632

**Published:** 2021-06-14

**Authors:** Antonio Moretta, Carmen Scieuzo, Anna Maria Petrone, Rosanna Salvia, Michele Dario Manniello, Antonio Franco, Donatella Lucchetti, Antonio Vassallo, Heiko Vogel, Alessandro Sgambato, Patrizia Falabella

**Affiliations:** ^1^ Department of Sciences, University of Basilicata, Potenza, Italy; ^2^ Spinoff XFlies s.r.l, University of Basilicata, Potenza, Italy; ^3^ Department of Translational Medicine and Surgery, Università Cattolica del Sacro Cuore, Rome, Italy; ^4^ Department of Entomology, Max Planck Institute for Chemical Ecology, Jena, Germany; ^5^ Centro di Riferimento Oncologico della Basilicata (IRCCS-CROB), Rionero in Vulture, Italy

**Keywords:** drug-resistant microorganisms, antimicrobial peptides, biomedical and pharmacological applications, pharmacokinetics and pharmacodynamics, drug delivery

## Abstract

Antibiotics are essential drugs used to treat pathogenic bacteria, but their prolonged use contributes to the development and spread of drug-resistant microorganisms. Antibiotic resistance is a serious challenge and has led to the need for new alternative molecules less prone to bacterial resistance. Antimicrobial peptides (AMPs) have aroused great interest as potential next-generation antibiotics, since they are bioactive small proteins, naturally produced by all living organisms, and representing the first line of defense against fungi, viruses and bacteria. AMPs are commonly classified according to their sources, which are represented by microorganisms, plants and animals, as well as to their secondary structure, their biosynthesis and their mechanism of action. They find application in different fields such as agriculture, food industry and medicine, on which we focused our attention in this review. Particularly, we examined AMP potential applicability in wound healing, skin infections and metabolic syndrome, considering their ability to act as potential Angiotensin-Converting Enzyme I and pancreatic lipase inhibitory peptides as well as antioxidant peptides. Moreover, we argued about the pharmacokinetic and pharmacodynamic approaches to develop new antibiotics, the drug development strategies and the formulation approaches which need to be taken into account in developing clinically suitable AMP applications.

## Introduction

A wide variety of antimicrobial agents are available today and they are broadly applied to treat different types of human infections. Specifically, antibiotics are powerful drugs used for treatments of pathogenic bacteria (Lei et al., 2019). However, their indiscriminate and prolonged use, especially in developing countries, in both human and veterinary medicine, as well as in agriculture have contributed to the development and spread of drug-resistant microorganisms (Huan et al., 2020). As the World Health Organization (WHO) has extensively announced, the alarming rise globally in resistance towards conventional antimicrobials represents a potential and serious risk to public health (Luong et al., 2020). Therefore, the antibiotic resistance issue has made it urgent to search for alternatives to conventional antibiotics, with novel modes of action and less predisposed to bacterial resistance. In the quest of new antibiotics, the antimicrobial peptides (AMPs), also known as host defense peptides, have recently raised great interest (Haney et al., 2019; Bhattacharjya and Straus, 2020; Mahlapuu et al., 2020). Current research is focused on these natural compounds as innovative anti-infective drugs and novel immunomodulatory candidates (Luong et al., 2020; Mahlapuu et al., 2020).

AMPs are bioactive small proteins, naturally produced by all living organisms as important and indispensable components of their innate immune system, becoming the first-line defense against microbial attacks in Eukaryotes, or produced as a competition strategy in Prokaryotes, to limit the growth of other microorganisms (Lei et al., 2019; Magana et al., 2020). Natural AMPs have potent and broad-spectrum activity against multiple classes of bacteria, yeasts, fungi, viruses and parasites (Huan et al., 2020; Luong et al., 2020), displaying bacteriostatic, microbicidal and cytolytic properties (Pasupuleti et al., 2012). Moreover, the interest in AMPs has recently increased during the Severe Acute Respiratory Syndrome Coronavirus 2 (SARS-CoV-2) pandemic in the search of new antiviral molecules to counteract COVID-19 disease (Kurpe et al., 2020).

AMPs were discovered in 1939, when the microbiologist René Dubos isolated from a soil *Bacillus* strain, an antimicrobial agent, named gramicidin, which was demonstrated to protect mice from pneumococcal infection (Van Epps, 2006). Afterwards, several AMPs have been discovered from both the prokaryotic and eukaryotic kingdom (Boparai and Sharma, 2020), including the tyrocidine, produced by the bacteria *Bacillus brevis*, with activity against bacteria, and the purothionin, identified in the plant *Triticum aestivum*, active against fungi and bacteria (Ohtani et al., 1977). The first described animal-originated AMP is defensin, which was isolated from rabbit leukocytes (Hirsch, 1956); subsequently lactoferrin was identified in cow milk (Groves et al., 1965) and it was demonstrated that lysosomes of human leukocytes (Zeya and Spitznagel, 1966) and human female reproductive tract contain low molecular weight AMPs (Sharma et al., 2011). To date, more than 3,000 AMPs have been discovered, characterized and annotated in the AMP database (APD3) (Huan et al., 2020), just considering that frog skin alone is a reservoir of more than 300 different AMPs (Boparai and Sharma, 2020).

### AMP Properties and Biosynthesis

Natural AMPs are evolutionary conserved gene-encoded molecules with structural and functional diversity, which is responsible for their wide range of activities against different pathogens in various organisms (Zhang and Gallo, 2016). However, although displaying considerable diversity in their physio-chemical and structural properties, origins and mechanisms of action, AMPs share some common features (Moravej et al., 2018). Indeed, they are mostly short molecules (<100 amino acids) (Pasupuleti et al., 2012), typically with a positive net charge (generally ranging from +2 to +11) and a notable proportion of hydrophobic residues (typically 50%) (Haney et al., 2017). They display an amphipathic structure, as they contain both hydrophobic and hydrophilic regions, that enable them to be soluble in aqueous environments (Boparai and Sharma, 2020). A less common class of AMPs is represented by the anionic AMPs, which have a negative net charge ranging from -1 to -7 and have been identified in vertebrates, invertebrates and plants (Harris et al., 2009). They include many negatively charged aspartic and glutamic acid residues, and in animals are found in various vital organs, including the brain, the epidermis, the respiratory and gastrointestinal tracts (Lakshmaiah Narayana and Chen, 2015). They show a different mechanism of action than the cationic ones. In order to facilitate their interaction with the target organism, some anionic AMPs use metal ions to form cationic salt bridges with negatively charged constituents of microbial membranes, allowing their penetration into the cell. When they reach the cytoplasm, they may attach to ribosomes or inhibit ribonuclease activity (Jeżowska-Bojczuk and Stokowa-Sołtys, 2018). Some anionic AMPs, such as theromyzin from *Theromyzon* tessulatum (Tasiemski et al., 2004), require zinc as a functional cofactor and it was found that the complex with zinc has stronger antimicrobial activity (Jiang et al., 2014).

Despite their relative similarity in biophysical characteristics, AMP sequences are rarely similar among closely related or distinct species/organisms (Pasupuleti et al., 2012). However, for some AMPs, a certain degree of identity is found either in the pro-region (the inactive sequence that is deleted by post-translational modifications) or in the amino acid patterns. This event could be due to species adaptation to the unique microbial environment that characterize the niche occupied by specific species (Pasupuleti et al., 2012).

**Figure 1:**
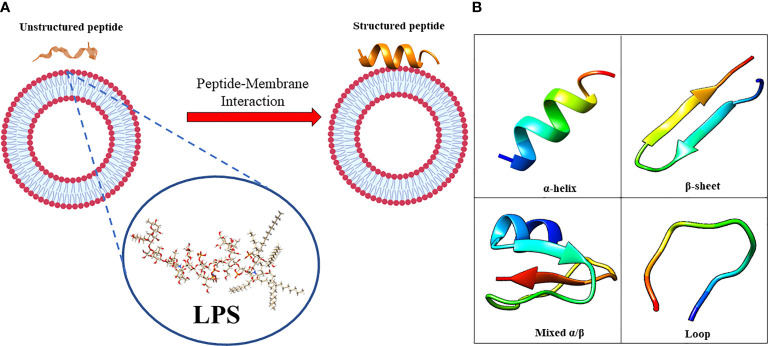
**(A)** in aqueous solution, the AMPs are unstructured while after the interaction with biological membrane, particularly with the LPS component, they assume the right conformation, which can be **(B)** α-helical, β-sheet, mixed α-helical/β-sheet, and loop. Figure created with Biorender.com and UCSF CHIMERA software (Pettersen et al., 2004).

The amphiphilic nature of the majority of AMPs is responsible for their structural flexibility. AMPs are commonly classified into four categories based on their secondary structure, including linear α-helical peptides, β-sheet peptides with the presence of 2 or more disulfide bonds, β-hairpin or loop peptides with the presence of a single disulfide bond and/or cyclization of peptide chain, and, finally, extended structures (Boparai and Sharma, 2020). Most AMPs belong to the first two categories. α-helical peptides display an unstructured conformation in aqueous solution but adopt an amphipathic helical structure in contact with biological membranes. However, a relevant feature is linked to the possible interactions with bacterial structures, such as lipopolysaccharides (LPS), that provoke conformational changes, influencing membrane permeabilization and the correct passage into the cytosol. Indeed, this interaction could change AMP tertiary structure, and AMP molecules could assume different conformations, such as monomeric helical or helix-loop-helix structures (**Figure 1**) (Bhunia et al., 2011).

For example, the contact with LPS induces oligomerization of specific AMPs, such as temporines, through the interaction among hydrophobic N and C terminal residues, preventing the correct movement throughout the membrane and the correct antimicrobial action (Bhunia et al., 2011). A particular amino acids composition could prevent this oligomerization, enhancing temporin activity. This is the case of temporin-1Tl, which is rich in aromatic residues with two positively charged amino acids (Bhunia et al., 2011). The synergy of temporin-1Tl with other temporins (Temporin A and Temporin B), prevent their oligomerization and facilitate the correct crossing of the bacterial membrane (Bhunia et al., 2011). Exceptions are related to some AMPs with particular structural characteristics, including the peptide MSI-594 (an analogue of magainin), that is unstructured in free solution, but have a folded helical hairpin structure when interact with LPS (Bhattacharjya, 2016). The interactions between two helical segments, facilitated by the fifth phenylalanine residue, allows the acquisition of the hairpin structure, implicating its very high activity against bacteria, fungi, and viruses (Domadia et al., 2010; Bhattacharjya, 2016). Another example of change in conformation after the interaction with LPS, is the β-hairpin structures of Tachyplesin I, that becomes more ordered and compact when interacting with LPS (Saravanan et al., 2012; Kushibiki et al., 2014). Another interesting example is linked to the human LL-37 AMP, one of the best studied peptides of this group, present in neutrophils and epithelial cells (Mahlapuu et al., 2016). It has been demonstrated that aromatic-aromatic interactions stabilize protein structure in correlation with lipids (Li et al., 2006) and that LL-37 could undergo a re-orientation depending on the concentration, suggesting also in this case an oligomerization process (Ding et al., 2013). On the contrary, β-sheet peptides are more ordered in aqueous solution because of their rigid structure and do not undergo radical conformational changes as helical peptides upon membrane interaction (Mahlapuu et al., 2016). It is not easy to clarify the structural conformations of β-sheet AMPs in membranes, because of the potential micelle aggregations; indeed, a recent report on thanatin peptide, isolated from insect *Podisus maculiventris*, showed dimerization of β-sheet structures (Sinha et al., 2017). These dimeric structures could facilitate the bond with LPS molecules, also at the distal ends, fostering bacterial cell associations and agglutination (Sinha et al., 2017). Defensins, a large group of AMPs, which are produced in macrophages, neutrophils and epithelial cells belong to this class (Mahlapuu et al., 2016). It was observed that the right combination of hydrophobicity, charge density and peptide length influence the antimicrobial activity of AMPs. Changing the amino acids position in the peptide chain or increasing the number of positively charged residues affect the secondary structure of AMPs, and consequently their biological activity against pathogens (Wu Q. et al., 2018). Besides the principle that the amino acid sequence determines the function of a peptide, it was found that the amino acid composition (in terms of abundance of residues with specific phyco-chemical properties) also affects AMP activity as clearly documented for a novel class of cationic AMPs known as “cationic intrinsically disordered antimicrobial peptides’’ or “CIDAMPs” since they are characterized by an intrinsically disordered structure. CIDAMPs have been detected in human skin and other barrier organs (Gerstel et al., 2018; Latendorf et al., 2019) and, carrying a positive net charge, have a low percentage of order-promoting amino acids (mostly hydrophobic residues commonly located within the hydrophobic core of foldable proteins) and a high percentage of disorder-promoting amino acids (mostly charged and polar residues, typically found at the surface of foldable proteins). They show microbicidal activity against several microbes, including *Candida albicans*, *Staphylococcus aureus* and *Pseudomonas aeruginosa* (Gerstel et al., 2018). The protein hornerin, expressed in the cornified epithelium, seems to be the main source of CIDAMPs, which act as disinfectants, helping to keep the surface of healthy skin free of infections (Gerstel et al., 2018).

AMP biosynthesis can occur in three different ways: classical ribosomal synthesis, non-ribosomal synthesis and proteolytic digestion of proteins (Buda De Cesare et al., 2020). Ribosomally synthesized AMPs, such as histatins and human β-defensins, are produced by ribosomal translation of specific mRNAs into the biologically active amino acid sequences in vertebrates, insects, plants, and bacteria. Non-ribosomally synthesized peptides are produced by large enzymes referred to as non-ribosomal peptide synthases, which incorporate non-proteinogenic amino acids into the sequence, and are found in filamentous fungi and bacteria (*Actinomycetes* and *Bacilli*). Finally, some AMPs, called cryptic peptides, are generated by proteolytic cleavage of bigger proteins with other functions. For example, the histone H2A of the Asian toad (*Duttaphrynus melanostictus*) is processed by the enzymatic activity of pepsin C producing buforin I, which in turn is processed by an endopeptidase to generate buforin II (Buda De Cesare et al., 2020). Interestingly, many AMPs are produced as inactive precursors and are active after proteolytic cleavage. Therefore, their activity is not only dependent on their own expression but also on the presence of appropriate proteases (Mahlapuu et al., 2016). The expression of AMPs can be constitutive or inducible by specific external factors (Mahlapuu et al., 2016; Lei et al., 2019). Some AMPs are expressed during the whole cellular lifetime but are stored at high concentration as precursors in granules and are released upon infection in the site of infection or inflammation (Mahlapuu et al., 2016). P9A and P9B are examples of inducible peptides, whose expression can be induced in silkmoth (*Bombyx mori*) hemolymph by vaccination with *Enterobacter cloacae*, as demonstrated by Hultmark and colleagues (Hultmark et al., 1980). In addition, Bals et al. (1999) reported that defensin production from epithelial cells of multiple mouse organs increases upon infection with *P. aeruginosa* PAO1.

### Insights Into the Mechanisms of Action of AMPs

**Figure 2:**
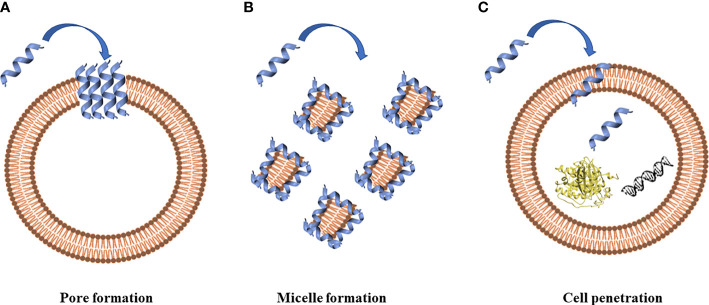
Antimicrobial peptides can act through a membranolytic and non-membranolytic mechanism. In the membranolytic mechanism AMPs can lead to **(A)** pore formation on the cell membrane or **(B)** micelle formation on the cell membrane. In the non-membranolytic mechanism, **(C)** AMPs can penetrate cell membranes and interact with intracellular targets, such as DNA and proteins. Figure created with Biorender.com and UCSF CHIMERA software (Pettersen et al., 2004).

The prerequisite to develop efficient AMPs as novel candidate drugs is the understanding of their mode of action. AMPs exert their activity by interaction with microbial cell membranes and this interaction is strongly affected by the lipid composition of biological membranes (Wu Q. et al., 2018). Since microbial membranes are the primary targets of AMPs, it is difficult for bacteria to develop resistance to AMPs as easily as to conventional antibiotics (Boparai and Sharma, 2020). Membrane interactions are mediated by electrostatic forces between positively charged AMPs and negatively charged microbial surfaces. The teichoic acids in the cell wall of Gram-positive bacteria and the LPS in the outer membrane of Gram-negative bacteria supply electronegative charge to the microbial surfaces, strengthening the interaction with AMPs (Boparai and Sharma, 2020). On the contrary, the outer layer of eukaryotic membranes is composed by zwitterionic phosphatidylcholine and sphingomyelin, which do not favor AMP interaction because of their neutral charge at physiological pH. Based on their mode of action, AMPs are divided into “membrane acting peptides”, which destabilize bacterial membranes causing their disruption, and “non-membrane acting peptides”, which are able to translocate across the membranes without damaging them but destabilizing normal cell functions (Boparai and Sharma, 2020) (**Figure 2**).

Three models have been proposed to explain the permeabilization of bacterial membranes by AMPs: barrel-stave model, toroidal-pore model and carpet model (Raheem and Straus, 2019). Thanks to their positive net charge, AMPs are able to interact with components of bacterial membranes, resulting in the disruption of the lipidic bilayer with cell death. AMP insertion can be perpendicular, as in the barrel-stave model, or perpendicular with the interaction with the head groups of lipids that provokes a deflection in the membrane (toroidal model) (Brogden, 2005). AMPs can also dispose parallel to the membrane, covering it completely, and forming, at the same time, micelles with the starting broken membranes (carpet model), as proposed by Gazit and colleagues in 1996 (Gazit et al., 1996). Moreover, defensins interact with LPS in Gram-negative bacteria and peptidoglycan in Gram-positive bacteria (Pachón-Ibáñez et al., 2017). Defensins have LPS-neutralizing activity in different bacteria (Lee et al., 2010) despite the chemical structure of LPS varies among them. LPS can self-aggregate forming oligomers above a Critical Micelle Concentration (CMC) because of its amphiphilic nature, a concentration of LPS, or any surfactant, above which it aggregates in micelles. It has been demonstrated that the association of defensin analogues and other peptides, such as gramicidin A, melittin, LL-37 and polymyxin B, with LPS leads to the disintegration of LPS aggregates. Moreover, it was observed that defensins amino acids (such as Arg, Trp, and Tyr) are involved in the stabilization of the peptide-pathogen surface complexes (Zhang et al., 2016).

The interaction with LPS has been demonstrated to be essential for AMPs like gramicidin S and polymyxin B to exert their mechanism of action for bacterial killing (Zhang et al., 2000). Bhunia and colleagues studied the structure of MSI-594 peptide in LPS micelles. They observed that the peptide is unstructured in solution, while it adopts a helix-loop-helix structure in complex with LPS, suggesting how AMPs could overcome the LPS barrier (Bhunia et al., 2009). A mutant form of MSI-594 peptide, substituting Phe5 with Ala amino acid, displays a limited permeabilization through the LPS layer suggesting that peptide conformation is essential to disrupt LPS (Domadia et al., 2010).

Other examples of AMPs acting by perturbation of microbial membrane structure are the fungal peptide alamethicin, the amphibian AMP aurein 1.2, and several defensins (Machado and Ottolini, 2015; Shahmiri et al., 2017; Su et al., 2018) AMPs acting through a non-membranolytic mechanism, thus displaying intracellular activities (such as inhibition of nucleic acids, proteins or cell wall synthesis), include buforin II and indolicidin that bind to DNA (Scocchi et al., 2016), teixobactin that binds to peptidoglycan precursor lipid II (Chiorean et al., 2020), Bac5 that interacts with ribosomes (Mardirossian et al., 2018) and Temporin-L, which binds FtsZ protein inhibiting *Escherichia coli* cell division (Di Somma et al., 2020). A recent study performed by Moura et al. demonstrated that the AMP thanatin interacts with LptC-LptA proteins, which belong to the Lpt complex, involved in the LPS transport, exploiting an inhibitory activity (Moura et al., 2020). Thanatin interaction with Lpt complex prevents LPS translocation to the outer membrane, modifying its stability and permeability and favoring the cell agglutination process (Dash and Bhattacharjya, 2021).

### Sources of AMPs and Their Potential Applications in Clinical Practice

The survival of organisms in an environment where pathogens are widely distributed, solely depends on their defense mechanisms. The inborn immunity of organisms involves endogenic peptides which supply a quick and viable method for safeguard against microbial attacks (Borah et al., 2020) AMPs are universal and essential components of the defense systems of all life forms, from bacteria to plants and invertebrate and vertebrate species, including mammals (Jenssen et al., 2006; Borah et al., 2020).

They are naturally produced in the body of both lower and higher organisms and their production is cell specific and may be constitutive or inducible in response to pathogenic challenges (Borah et al., 2020). In multicellular organisms, AMPs are mostly localized to specific sites that are normally more exposed to microbes, such as the skin and mucosa epithelia (Jenssen et al., 2006). The primary role of these defense peptides is the killing of invading pathogens; however, in higher organisms they act also as modulators of the innate immune response (Jenssen et al., 2006). AMPs are commonly classified according to their sources, which are represented by microorganisms, plants, and animals.

Below, we give an overview of various naturally occurring AMPs and the potential clinical application of some of them.

#### Microorganisms as Source of AMPs

Bacteria and fungi are reservoirs of AMPs (Huan et al., 2020). Among the numerous AMPs, the first isolated and characterized were those produced by bacteria (Jenssen et al., 2006). AMPs from bacteria are not produced for the purpose to protect against infections, but rather as a competition strategy (Jenssen et al., 2006). With their activity they kill other microbes competing for nutrients in the same niches, ensuring the survival of individual bacterial cells (Jenssen et al., 2006). Bacterial AMPs, also called bacteriocins, are represented by a heterogeneous family of small ribosomally synthesized molecules with strong antimicrobial activity at specific concentrations (Soltani et al., 2021). These molecules, produced by Gram-positive and Gram-negative bacteria, are effective against many pathogenic bacteria and are extraordinarily active compared to their eukaryotic counterparts (Jenssen et al., 2006; Soltani et al., 2021). For example, AMPs isolated from *Pseudomonas* spp display activity against several bacterial species, such as *S. aureus*, *E. coli*, *Salmonella*, *Shigella*, showing both general antibacterial and specific antibiofilm activity (Fontoura et al., 2008; Mohammadi-Barzelighi et al., 2019). Mersacidin, isolated by *Bacillus* spp, shows *in vivo* bactericidal activity against Methicillin-resistant *S. aureus* (MRSA) equivalent to that of vancomycin (Jenssen et al., 2006).

AMPs are also produced by human microbiota. Host-microbiota crosstalk is based on AMPs secretion by phagocytic and epithelial cells and microbiota of the human gut, skin, and oral cavity; these peptides contribute to microbial and ecological balance (Magana et al., 2020). An example of these human microbiota AMPs is the thiopeptide lactocillin produced by the vaginal commensal *Lactobacillus gasseri* and acting against Gram-positive bacteria, including *S. aureus* and *Gardnerella vaginalis* (He et al., 2020).

Several filamentous fungi produce AMPs which are similar to plant and animal defensins. Examples of cysteine-rich defensin-like AMPs in ascomycetes are AFP from *Aspergillus giganteus*, PAF from *Penicillium chrysogenum*, ANAFP from *Aspergillus niger*, AcAFP and AcAMP from *Aspergillus clavatus* (Montesinos, 2007; Hegedüs and Marx, 2013). All these fungal peptides have antifungal activity against filamentous ascomycetes, including animal and plant opportunistic and pathogens, such as *Aspergillus fumigatus*, *Fusarium* sp., and *Botrytis* sp. (Hegedüs and Marx, 2013).

On the basis of their antimicrobial properties and their safety and tolerability, some of these natural AMPs have potential therapeutic applications. The bacteriocin nisin, produced by *Lactococcus lactis*, has been extensively studied being used as food preservative (Soltani et al., 2021). Nisin is the only bacteriocin legally approved as biopreservative and is used in the dairy industry to control contamination from *Listeria* strains (Soltani et al., 2021). Because of its broad-spectrum activity against both Gram-positive and Gram-negative pathogens, nisin is approved for clinical use as an alternative to antibiotics (Dijksteel et al., 2021). Several studies have reported the suitability of nisin in the treatment of several infection diseases, such as mastitis (Cao et al., 2007; Fernández et al., 2008), oral (Shin et al., 2015; Mitra et al., 2019), respiratory (De Kwaadsteniet et al., 2009) and skin (Heunis et al., 2013) infections. Johnson et al. (1978) have been the first to demonstrate that there were fewer numbers of *streptococci* in the dental plaque of monkeys that received nisin in their foods. Moreover, more recent studies support the antimicrobial abilities of nisin against oral pathogenic bacteria relevant to periodontal diseases and caries. Indeed, Tong et al. (2010) showed that nisin A is able to inhibit the growth of cariogenic bacteria. Cao et al. (2007) demonstrated that a nisin‐based formulation was effective in the treatment of clinical mastitis in lactating dairy cows caused by different mastitis pathogens. Mastitis is a common inflammatory disease in lactating women, which causes breastfeeding cessation (Foxman et al., 2002). *S. aureus* and *Staphylococcus epidermidis* are two common agents that cause mastitis‐associated infections (Foxman et al., 2002). Nisin peptide causes bacterial growth inhibition by membrane pores formation and by interrupting the cell wall biosynthesis through specific lipid II interaction (Prince et al., 2016).

Another example of bacterially derived AMPs used in clinics as alternative to antibiotics is gramicidin, which is a mix of gramicidin A, B and C. They are AMPs naturally produced by *Bacillus brevis*, with activity against several Gram-positive bacteria, inducing membrane depolarization and consequently cell lysis (David and Rajasekaran, 2015; Yang and Yourself, 2018). Gramicidin is a constituent of Neosporin^®^, a triple antibiotic used in ophthalmic and topical preparations (Hallett et al., 1956). Gramicidin S is used in the treatment of wound infection and of the root canal of teeth due to the tetracycline resistant *Enterococcus faecalis* biofilms formation (Berditsch et al., 2016). The bacterium *Streptomyces roseosporus* is a rich source of the anionic AMP daptomycin, which shows bactericidal activity against Gram-positive pathogens (Ball et al., 2004). Daptomycin exerts its bactericidal action by formation of membrane pores, membrane depolarization and inhibition of cell wall synthesis (Taylor and Palmer, 2016). This peptide has been approved and marketed as anionic AMP for the treatment of skin infections caused by Gram-positive bacteria (Wang et al., 2014).

Considering the great variety of AMPs existing in nature, it has to be expected that other novel nature-inspired peptides, pharmacological active, might find clinical applications in the future.

#### Plants as Source of AMPs

Bioactive peptides are essential components of plants defense mechanisms, with extraordinary physiological importance, providing fast protection against bacterial and fungal infections (Jenssen et al., 2006; López-Meza et al., 2011; Salas et al., 2015). Plant AMPs not only display microbicide activities but are also involved in cellular signaling (Salas et al., 2015). Several active peptides have been extracted and isolated from roots, flowers, seeds, stems and leaves and are classified based on their amino acids sequence, position and number of cysteine residues involved in the disulfide bridge formation (López-Meza et al., 2011). Ten families of plant AMPs have been described (López-Meza et al., 2011) and the best-studied groups are defensins, thionins and snakins (Jenssen et al., 2006; López-Meza et al., 2011; Huan et al., 2020). The first plant-derived AMP is purothionin, which displays activity against *Corynebacterium fascians*, *Pseudomonas solanacearum*, *Corynebacterium poinsettia* (de Caleya et al., 1972). Plant defensins are cysteine-rich AMPs, with four disulphide bridges and a globular structure (Salas et al., 2015); they are basic peptides, composed by 45 to 54 amino acid residues, ubiquitous in the plant kingdom, displaying activities against bacteria and fungi. The PvD1 peptide is a defensin from *Phaseolus vulgaris*, which inhibits growth of yeasts, such as *Candida albicans*, *Candida tropicalis* and *Saccharomyces cerevisiae* (Mello et al., 2011). Thionins, composed by 45 to 47 amino acids, are basic peptides found in several plant tissues, which are toxic to bacteria and phytopathogenic fungi (López-Meza et al., 2011). Snakins are small peptides with 12 cysteine residues forming six disulphide bridges, essential for their biological activity (Meneguetti et al., 2017). Snakin-Z from *Ziziphus jujuba*, composed by 31 amino acids, is more toxic for fungi than bacteria (Meneguetti et al., 2017). Finally, different AMPs have been identified in avocado fruit and in fruits of *Capsicum*, which for their antimicrobial properties could be used in the treatment of infections caused by *S. aureus* and *E. coli* strains (Liu et al., 2006; Guzmán-Rodríguez et al., 2013; Taveira et al., 2014).

Considering their efficiency and broad-spectrum activity, plant AMPs may represent a promising alternative to conventional antibiotics for counteracting infections (da Silva and Machado, 2012).

#### Animals as Source of AMPs

Animal AMPs are produced at the sites that are constantly exposed to microbes, such as skin and mucosal barriers (López-Meza et al., 2011). Various AMPs have been isolated from invertebrates and many vertebrate species (including fish, amphibians, and mammals).

In invertebrates the innate immune system is extremely efficient since they lack an adaptive immune system, and in this regard, AMPs play a key role in protection against foreign microbial attacks (Jenssen et al., 2006). Invertebrates can produce a wide range of proteins and peptides which are found in phagocytes, in epithelial cells and in hemolymph (plasma and hemocytes) (Jenssen et al., 2006). The β-hairpin-like peptides tachyplesin (Nakamura et al., 1988) and polyphemusin (Miyata et al., 1989) (from horseshoe crab), and melittin (from bee venom) (Raghuraman and Chattopadhyay, 2007) are examples of invertebrate AMPs.

A recent study has demonstrated that a pretreatment with Tachyplesin III on mice protects them against *P. aeruginosa* and *Acinetobacter baumannii* infection, reduces the production of pro-inflammatory cytokines (IL-1β, IL-6, and TNF-α) and induces the macrophage phagocytosis, fundamental to exert bacterial clearance, in a dose-dependent manner (Qi et al., 2019). All these findings must be confirmed in human clinical trials.

More than 200 AMPs have been isolated in insects (Li et al., 2012). The number of these bioactive molecules varies between species. *Hermetia illucens* and *Harmonia axyridis* produce up to 50 AMPs, while they are not found in other species, such as *Acyrthosiphon pisum* (Huan et al., 2020; Moretta et al., 2020). AMPs are produced mainly in the fat body and blood cells (hemocytes) of insects and then are secreted into the hemolymph (Jenssen et al., 2006; Huan et al., 2020). Based on their amino acid sequences and antimicrobial activities, insect AMPs are divided into several groups: cecropins, defensins, proline-rich and glycine-rich peptides (Manniello et al., 2021). Cecropin was the first insect AMP discovered in the hemolymph of the pupae of *Hyalophora cecropia* (Steiner et al., 1981). Cecropins, which are described only in the order *Diptera* and *Lepidoptera*, are linear peptides with α-helix and without cysteines, composed by around 35 amino acid residues and displaying activity against Gram-positive and Gram-negative bacteria (Wu Q. et al., 2018). Insect defensins are inducible peptides which display strong activity against Gram-positive bacteria and less against Gram-negative bacteria. They are composed by 29-34 amino acid residues and have been isolated from several insect orders, such as Coleoptera, Hemiptera Diptera, Trichoptera, Hymenoptera and Odonata (Bulet et al., 1999). Attacins are an example of glycine-rich AMPs, which show activity against Gram-negative bacteria, including *E. coli* (Carlsson et al., 1991). This group of peptides is heterologous in size, but their common feature is the high content of glycine-residues (10-22%) (Wu Q. et al., 2018), which affect the tertiary structure and consequently their mode of action (Li et al., 2012). Diptericin, Coleoptericin, Sarcotoxin IIA are other glycine-rich AMPs isolated from insects (Ando and Natori, 1988; Dimarcq et al., 1988; Sagisaka et al., 2001). Although insect AMPs could be a good alternative to conventional antibiotics, their clinical use is still limited and most of them are just *in vitro* tested (Manniello et al., 2021).

Among them, the melittin peptide is, currently, in clinical use for its antimicrobial potency. Composed by 26 amino acids, melittin is the principal component of venom from the honeybee *Apis mellifera*. Melittin has broad spectrum activity, and its ability to protect *in vivo* against MRSA infections has been demonstrated (Choi et al., 2015). It acts by induction of pore formation following interaction with membrane surfaces (van den Bogaart et al., 2008). Since it also shows anti-inflammatory properties (Lee and Bae, 2016), the Food and Drug Administration (FDA) approved its usage in clinical practice (Dijksteel et al., 2021), for relieving pain associated to tendinitis, arthritis, sclerosis multiple (Park et al., 2004; Son et al., 2007; Yang et al., 2011).

Amphibians, especially frogs, are a rich source of AMPs. Most of the amphibian AMPs have been isolated from the frog skin. These biologically active molecules are released from cutaneous glands and excreted towards the skin surface following pathogen stimulations (Patockaa et al., 2018). The prototypic and the most famous AMP from frogs is the α-helical magainin (Zasloff, 1987), which is active against yeasts, fungi, bacteria, and viruses (Borah et al., 2020). Esculentins, nigrocins, brevinins, temporins are some of the best characterized peptides produced by frogs of the genus *Rana* (Patockaa et al., 2018). The basic esculentin-1 peptide, composed by 46 amino acid residues and a disulphide bridge, exhibits strong activity against several human pathogens, such as *C. albicans*, *P. aeruginosa*, *E. coli* and *S. aureus* (Patockaa et al., 2018).

Esculentin was *in vitro* tested on human lung epithelium to determine the toxicity, finding a good tolerability in terms of inflammatory effects. Then, it was studied in a mouse model, in which a lung-infection was induced with *P. aeruginosa*: promising results showed a strong reduction in bacterial load not only in lungs but also in spleen, indicating a decrease in systemic spread of bacteria (Chen C. et al., 2017).

Brevinin-2Ta was tested on mice infected with *Klebsiella pneumoniae.* In this study, it was demonstrated that the peptide decreases the bacterial load, altering the microorganism structures in infection sites and it also showed the ability to faster angiogenesis and granulation tissue maturing process, obtaining comparable results to classical antibiotics. For this reason, this peptide is a good candidate for pre-clinical studies, even if some modifications are needed in order to decrease its hemolytic power (Liu et al., 2017). Liu et al. (2017), hypothesized that amino acid substitutions in the primary structure could be the right strategy to reduce the hemolytic activity, improving, at the same time, the antimicrobial one.

Regarding anionic AMPs, the temporin-1Ja, carrying a net charge of -1, has been isolated from the skin secretions of the Japanese frog *Rana japonica* (Isaacson et al., 2002). This anionic peptide revealed moderate activity against *E. coli* and *S. aureus* strains. However, it was found that this peptide synergizes with other temporins, contributing to endotoxin neutralization (Rosenfeld et al., 2006). AMPs can also protect amphibians from ingested pathogens since they are produced in the mucosa of the stomach. The Asian toad peptide buforin and buforin II are the best characterized examples in this regard (Jenssen et al., 2006). Some of these natural AMPs have been used for the production of synthetic peptides, such as the Pexiganan, also known as MSI-78. It is a synthetic 22-amino-acid analogue of magainin–2, which has been tested as a topical cream for treatment of bacterial infections related to diabetic foot ulcers. It showed promising *in vitro* broad-spectrum activity (Ge et al., 1999), but it was rejected by FDA because there was no advantage compared to conventional antibiotics (Koo and Seo, 2019).

Mammalian AMPs have been identified in humans, cattle, sheep and other vertebrates (Huan et al., 2020). Some AMPs from mammalians have a second major function inducing chemoattraction and activation of host cells to engage in innate host defense (Yang et al., 2001). AMPs can be stored in phagocytes and epithelial cells and can be released extracellularly by degranulation in response to different stimuli, becoming available at the site of infection (Yang et al., 2001). For example, cathelicidins are stored within granules of circulating immune cells as inactive propeptides (Jenssen et al., 2006). Cathelicidins and defensins are the main AMPs found in mammalians, such as humans, horses, rabbits, sheep and mice. Cathelicidin family comprises heterogeneous peptides which share the N-terminal pro-region but show a variable antibacterial peptide in the C-terminal region, displaying different structures, including β-hairpin, α-helical, and arginine and proline-rich peptides (Kościuczuk et al., 2012). This structural diversity reflets cathelicidin different functions and their diverse spectrum of antimicrobial and immunomodulatory activities (Jenssen et al., 2006). The α-helical BMAP-28 is a bovine AMP of the cathelicidin family which is able to permeabilize the membranes of several bacteria and fungi at a moderate concentration *in vitro* (Risso et al., 2002; Benincasa et al., 2006). Only one cathelicidin, the hCAP18 (better known as LL-37), is produced in humans and has been isolated from specific granules of neutrophil granulocytes. A second group of mammalian AMPs are the defensins, which require proteolytic processing to acquire their active form (Selsted and Ouellette, 2005). More than 50 defensins have been identified in mammalian species; some of them are stored in granules of macrophages, neutrophils and Paneth cells, while others are produced by mucosal epithelial cells and keratinocytes (Yang et al., 2001). Defensins production can be constitutive, such as for human β-defensin-1 (hBD1), or inducible, such as for hBD2, whose expression is induced by exposure to bacteria or microbial components, as LPS (Jenssen et al., 2006). Maiti et al. (2014) studied mice mortality after the infection with *Salmonella typhimurium*, demonstrating that the administration of hBD1, hBD2, or a combination of both, lead to an increased mice mortality and a decreased *S. typhimurium* load in peritoneal fluid, liver and spleen.

The anionic peptide Dermcidin, discovered in epithelial and neutrophil granules of humans, is one of the most studied human anionic AMPs. This peptide is proteolytically processed in sweat producing several truncated peptides which display a good spectrum of antimicrobial activity (Schittek et al., 2001).

There are several examples of mammalian AMPs proposed for clinical applications. The acid-pepsin digestion of bovine lactoferrin results in the release of the peptide lactoferricin, which shows the strongest antimicrobial activity among mammalian lactoferricins (Vorland et al., 1998) and has potent immunological and antitumor properties (Gifford et al., 2005; Yin et al., 2013; Arias et al., 2017). It exerts its bactericidal activity on Gram-positive and Gram-negative bacteria inducing depolarization of the cell membrane, with fusion of negatively charged liposomes and formation of blebs on the cell surface (Ulvatne et al., 2001; Bruni et al., 2016). The bovine lactoferricin displays useful properties for potential applications in human medicine. It has been successfully utilized for treatment of enterohemorrhagic *E. coli* infections (Kühnle et al., 2019). Because of its antimicrobial and anti-inflammatory properties, the bovine lactoferricin can be used for treatment of ocular infections, since it potentiates the effect of conventional antibiotics against clinical ocular isolates of *P. aeruginosa* and *S. aureus* (Oo et al., 2010). Moreover, it improves diabetic wound healing (Mouritzen et al., 2021) and finds applications in the treatment of osteo-articular diseases (Yan et al., 2013). The saliva of humans and other primates contains various forms of AMPs, among them the histatins, which are small histidine-rich cationic peptides with antifungal properties. Histatin 5, that is the product of histatin 3 proteolytic cleavage, is the most active histatin against several yeasts, such as *Cryptococcus neoformans*, *Candida dubliniensis* and *Candida albicans* (da Costa et al., 2015). Histatins exert their activity by targeting the mitochondria, affecting cell respiration (Kavanagh and Dowd, 2004) and, because of their safety and tolerance, have been successfully tested in topical gels to treat oral fungal infections (Paquette et al., 2002). Several efforts have been made to identify fragments of histatin 5 with pharmaceutical application and have yielded promising results. An example in this regard is the 12-amino acid peptide P113, which was evaluated in phase I and phase II clinical studies as pharmaceutical agent to fight oral candidiasis (Woong et al., 2008; Cheng et al., 2018; Browne et al., 2020).

**Tables 1** and **2** summarize, respectively, naturally occurring AMPs from different sources and those used in clinical practice.

**Table 1:** Overview of AMPs from different sources in nature and the current status of research.

AMPs from Microorganism					
Class	Source	Peptide Name	Biological activity	Studies	Reference
Bacteriocin	Bacteria *Bacillus* spp.	Mersacidin	Antibacterial	*In vivo*	Jenssen et al., 2006 Kruszewska et al., 2004
Bacteriocin	Bacteria *Lactobacillus gasseri*	Lactocillin	Antibacterial	*In vitro*	Magana et al., 2020 Donia et al., 2014
Bacteriocin	Bacteria *Lactococcus lactis*	Nisin	Antibacterial	Clinical practice	Dijksteel et al., 2021
Bacteriocin	Bacteria *Bacillus subtilis*	Ericin	Antibacterial	*In vitro*	Sharma et al., 2018
Defensin	Fungi *Penicillium chrysogenum*	PAF	Antifungal	*In vivo*	Kaiserer et al., 2003 Barna et al., 2008 Marx, 2004 Palicz et al., 2016
Defensin	Fungi *Aspergillus giganteus*	AFP	Antifungal	*In vitro*	Hegedüs and Marx, 2013 Krishnamurthy et al., 2020
**AMPs from Plants**					
Defensin	*Phaseolus vulgaris*	PvD1	Antifungal	*In vitro*	Mello et al., 2011 do Nascimento et al., 2015
Defensin	Persea americana	*Pa*Def	Antibacterial	*In vitro*	Guzmán-Rodríguez et al., 2013
Thionin	*Triticum aestivum*	α1-purothionin	Antibacterial	*In vitro*	de Caleya et al., 1972 Oard et al., 2012
Snakin	*Ziziphus jujuba*	Snakin-Z	Antifungal	*In vitro*	Daneshmand et al., 2013 Meneguetti et al., 2017
**AMPs from Insects**					
Cecropin	*Hyalophora cecropia*	CecA	Antibacterial	*In vitro*	Wu Q. et al., 2018 Wang et al., 2017
Cecropin	*Spodoptera litura*	Spodopsin Ia	Antibacterial	Discovery	Choi et al., 1997
Defensin	*Drosophila melanogaster*	Drosomycin	Antifungal	*In vitro*	Landon et al., 1997 Fehlbaum et al., 1994
Proline-rich AMPs	*Apis mellifera*	Abaecin	Antibacterial	*In vitro*	Casteels et al., 1990 Luiz et al., 2017
Attacin	*Hyphantria cunea*	Attacin-B	Antibacterial	*In vitro*	Kwon et al., 2008
Glycine-rich AMPs	*Drosophila melanogaster*	Diptericin	Antibacterial	*In vitro*	Verma and Tapadia, 2012 Wicker et al., 1990
**AMPs from Animals**					
Cathelicidin	Bovine	BMAP-28	Antibacterial	*In vivo*	Risso et al., 2002 Benincasa et al., 2003
Brevinin	*Rana boylii*	Brevinin-1BYa	Antifungal	*In vivo*	Conlon et al., 2003 Liu et al., 2017
Cathelicidin	Pig	Protegrin-1	Antibacterial	*In vitro*	Soundrarajan et al., 2019 Huynh et al., 2018
**AMPs from Humans**					
Cathelicidin	Human granulocytes	hCAP18/LL-37	Antibacterial	Clinical trial	Leszczynska et al., 2013
Defensin	Human monocytes	hBD1 hBD2 hBD3	Antibacterial	*In vivo*	Levón et al., 2015 Maiti et al., 2014
Histatin	Human saliva	Histatin-1	Antibacterial Antifungal	Clinical practice	Khurshid et al., 2017

**Table 2:** List of natural AMPs in clinical practice.

Peptide Name	Origin	Mechanism of action	Indication	Reference
Nisin	Bacteria (*Lactococcus lactis*)	Membrane depolarization	Bacterial infections	Cao et al., 2007 Mitra et al., 2019
Gramicidin	Bacteria (*Brevibacillus brevis*)	Membrane depolarization/Lysis	Bacterial conjunctivitis	David and Rajasekaran, 2015
Melittin	Insect (*Apis mellifera*)	Membrane disruption	Anti-inflammatory applications	Lee and Bae, 2016
Daptomycin	Bacteria (*Streptomyces roseosporus*)	Membrane depolarization/Lysis	Skin infections	Taylor and Palmer, 2016
Lactoferricin	Mammalians	Membrane depolarization	Anti-inflammatory applications	Oo et al., 2010 Yan et al., 2013
Histatin	Humans	Inhibition of respiration	Fungal infections	Paquette et al., 2002

## AMPs: Innate Weapons Against Diseases

Given the broad spectrum of action of the AMPs, their diversity in sequences and considering the physico-chemical characteristics related to their several sources, they can find application in different fields. Specifically, below we addressed the suitability of AMPs in the biomedical and pharmacological fields, also taking into account the pharmacokinetic and pharmacodynamic approaches to develop new molecules with antimicrobial activity.

The excessive use of antibiotics in clinical treatment has increased pathogens resistance to these compounds (Aminov, 2010). The pharmaceutical industry is trying to solve this problem by looking for new molecules with antibiotic activity or by modifying/improving the existing ones. Nevertheless, pathogens can develop resistance mechanisms that compromise this strategy. Thus, the need to find new active molecules with different mechanisms of action represents one of the most urgent challenges in medicine (Parisien et al., 2008). AMPs are among the most promising alternatives to modern antibiotics and they have already found clinical applications in this field, as previously mentioned, alone or in synergy with existing antibiotics. AMPs are susceptible to proteolysis due to their chemical characteristics and their activity is affected by salts concentration and pH. For this reason, the most promising applications for AMPs in clinical evaluations are those involving topical applications (Hancock and Sahl, 2006). The endogenous production of AMPs is also relevant and worth further studies. For example, sodium butyrate administration has been shown to induce the production of intestinal AMPs, beneficial for the treatment of infectious or inflammatory diseases (Guaní-Guerra et al., 2010).

However, AMPs broad spectrum of biological activities suggests other potential clinical benefits such as for the treatment of cancer and viral infections as well as in the immune system modulation (Schweizer, 2009).

### Involvement of AMPs in Respiratory Diseases

Infections in the lower respiratory tract are involved in chronic inflammatory lung disorders such as cystic fibrosis and chronic obstructive pulmonary disease. In cystic fibrosis patients with a *P. aeruginosa* infection, this organism produces AMPs, such as pyocins, which inhibit the growth of its closest competitors. Thus, the same AMPs could be used as a therapeutic agent to minimize the effects of the infection, besides rooting out other susceptible pathogens. Pyocins derived from *P. aeruginosa* strains also have toxic effects on *Haemophilus*, *Neisseria* and *Campylobacter* strains and have been successfully used for the treatment of peritonitis in mice (Scholl and Martin, 2008; Waite and Curtis, 2009).

It is of interest that neutrophils and airway epithelial cells produce AMPs to prevent infection of the respiratory system by pathogens. In cystic fibrosis patients, *P. aeruginosa* induces the secretion of sPLA2-IIA by airways epithelial cells *via* a Krüppel-like transcription factor (KLF)-2-dependent pathway, that lead to the selective death of *S. aureus* (Rahnamaeian, 2011).

Moreover, the serum level of the human LL-37 peptide is higher in patients with lower respiratory tract infections than in healthy people (Majewski et al., 2018). Recently, it has been reported that the Esculentin peptide (1−21), active on both *P. aeruginosa* planktonic and biofilm forms, has the ability to prolong the survival of mouse models with pulmonary infection. The main AMPs detected in lung tissues and secretions of cystic fibrosis patients are sPLA2-IIA, neutrophil α-defensins/HNPs, hBDs and LL-37 (Hiemstra et al., 2016).

Similar phenomena have been described in periodontal diseases caused by *Porphyromonas gingivalis* in which the sPLA2-IIA peptide is produced by oral epithelial cells *via* activation of the Notch-1 receptor and kills oral bacteria (Balestrieri et al., 2009).

### AMPs in Wound Healing and Skin Infections

Skin and soft tissue infections are one the most common microbial infections in humans and AMPs can be a new therapeutic option thanks to their broad-spectrum of biological activities, since skin pathogens include bacteria but also protozoa, fungi and viruses (Sunderkötter and Becker, 2015). Moreover, AMP preparations have the advantage of high concentration at the target site for topical administration because of their low ability to penetrate into the bloodstream. Moreover, AMPs can promote wound healing by modulating cell migration, angiogenesis, chemotaxis, and cytokine release (Ramos et al., 2011).

For example, the hBD2 is induced by the Epidermal Growth Factor Receptor (EGFR) activation and it can increase keratinocyte migration and cytokines production (Sørensen, 2016). Another peptide highly expressed by keratinocytes at wound sites is represented by hBD3 defensin. It promotes cytokine secretion, cell migration and proliferation by phosphorylating EGFR and STAT proteins (Sørensen et al., 2005). It also speeds up the wound closure when topically applied in a porcine model of infected skin wounds (Hirsch et al., 2009). Moreover, it has been demonstrated that hBD3 exhibits anti-inflammatory activity through the inhibition of TLR (Toll-like receptor) signaling pathways in immune cells leading to a transcriptional repression of the pro-inflammatory genes (Semple et al., 2011).

The expression of skin LL-37 peptide is also increased after wounding (Heilborn et al., 2003), and it seems to be involved in the modulation of angiogenesis. Indeed, LL-37 peptide stimulates endothelial cells proliferation and neovascularization by activating the formyl peptide receptor-like 1 (FPR2/ALX) (Koczulla et al., 2003).

Psoriasis vulgaris is an inflammatory skin disease characterized by abnormal epidermal proliferation and a cellular infiltrate including neutrophils and T cells (Davidovici et al., 2010). Due to the enhanced proliferation rate of psoriatic keratinocytes associated with a reduction of the cell cycle duration, psoriasis has been thought to be an epidermal disease. However, experiments performed with severe combined immunodeficiency (SCID) mice indicated that psoriatic eruptions are induced by CD4+ cells and T cells are believed to play a key role in the pathogenesis of psoriasis (Ellis et al., 1986; Wrone-Smith and Nickoloff, 1996).

The keratinocytes within the epidermis of psoriatic plaques are abnormal and among the abnormalities there is the excessive production of AMPs which, in vertebrates, are believed to modify host inflammatory responses through different mechanisms including regulation of cell proliferation, chemotactic and angiogenic activities (Lai and Gallo, 2009).

HNP1, HNP2, HNP3, hBD2 and hBD3 are defensins identified from lesional psoriatic scale extracts and their presence could help to explain why a hyperproliferative and noninfectious skin disease, such as psoriasis, undergoes less cutaneous infections than it would be expected (Harder et al., 2001; Harder and Schröder, 2005). Studies performed on LL-37 peptide demonstrated that it has both pro-inflammatory and anti-inflammatory activity, can promote chemotaxis, angiogenesis and enhance wound repair (Yang et al., 2000; Koczulla et al., 2003; Braff et al., 2005; Tokumaru et al., 2005; Mookherjee et al., 2006). Frohm *et al.* were the first to report that cathelicidin/LL-37 expression is upregulated in psoriatic epidermis and suggested that this induction increases the antimicrobial defense ability of the disrupted barrier in the lesions (Frohm et al., 1997). Later, it has been hypothesized that LL-37 could drive inflammation in psoriasis by allowing plasmacytoid dendritic cells (pDCs) to recognize self-DNA through TLR9 (Lande et al., 2007).

### Angiotensin-Converting Enzyme I (ACE) Inhibitory Peptides

The angiotensin-converting enzyme I (ACE) is produced by lung or kidney tissue and the luminal membrane of vascular endothelial cells. ACE converts inactive decapeptide angiotensin I (ANG I) into vasoconstrictor octapeptide angiotensin II (ANG II). ANG II is involved in several physiological and pathophysiological cardiovascular conditions such as atherosclerosis and hypertension (Wu C. H. et al., 2018). ACE inhibitors are used in hypertension treatment, but they may cause serious side effects, such as cough, rush and edema (Wu C. H. et al., 2018). Hence, it derives the need to identify new and nontoxic ACE inhibitors, whose activity depends on the amount and type of amino acid composition.

It has been observed that the binding to ACE is influenced by hydrophobic amino acids at the peptide C-terminus (Salampessy et al., 2017). Moreover, amino acids like alanine, valine, isoleucine, isoleucine and glycine – which are hydrophobic residues with aliphatic side chains – at the C-terminus have been associated with an increase in the ACE inhibitory activity (Toopcham et al., 2017). SAGGYIW and APATPSFW are two AMPs able to act as ACE inhibitors potentially suitable as antihypertensive peptides. They are produced in wheat gluten hydrolysate by the *P. aeruginosa* protease and contain tryptophan at the C-terminus (Zhang et al., 2020). This observation led to the idea that the presence of a tryptophan at the C-terminus of a peptide could influence the ACE inhibitory activity by blocking the enzyme active site *via* weak interactions, such as electrostatic, hydrophobic and Van Der Waals interactions and hydrogen bonds.

Another example is the VEGY peptide, which was isolated from the marine *Chlorella ellipsoidea* and has been demonstrated to exhibit ACE inhibitory activity and to be stable against gastrointestinal enzymes (Ko et al., 2012). This potential use of AMPs certainly represents a fruitful avenue of pursuit and will likely find clinical applications in the future.

### Pancreatic Lipase Inhibitory Peptides

Obesity and fatty acid metabolism disorders are widespread epidemic. One of the pharmacological strategies to counteract these issues is the dietary lipid inhibition. The pancreatic lipase enzyme hydrolyzes 50–70% of food-derived fat in the human organism and its inhibition is exploited by the Orlistat drug used in obesity treatment. However, in long-term treatment, this strategy can cause side effects, such as pancreatic damage and gastrointestinal toxicity (Cheung et al., 2013). For this reason, the search of new compounds able to inhibit pancreatic lipase, without exerting side effects, represents a still alive need to fight these disorders. Several AMPs have been identified so far that are able to show this activity, which depends on the structure and amino acid composition of the peptide (Hüttl et al., 2013). CQPHPGQTC, EITPEKNPQLR and RKQEEDEDEEQQRE are three peptides from purified soybean β‐conglycinin that have been demonstrated to inhibit the pancreatic lipase (Lunder et al., 2005; Martinez-Villaluenga et al., 2010), and are under investigation for potential clinical applications (Złotek et al., 2020).

### Peptides With Antioxidant Activity

Oxidative stress, caused by an imbalance between production and removal of reactive oxygen species (ROS) in cells and tissues, can promote diseases like obesity, diabetes, and heart disease (Pizzino et al., 2017). Environmental stressors like pollutants, heavy metals, xenobiotics, high-fat diet and the progression of aging can contribute to an increase in ROS production. Oxidative stress is also involved in several neurological disorders such as Alzheimer’s and Parkinson’s diseases (Singh et al., 2019).

A growing number of antioxidant AMPs have been identified from different sources, including animals, plants and insects (Balti et al., 2010; Villadóniga and Cantera, 2019; Liang et al., 2020). Peptide antioxidant activity is related to their sequence and amino acid composition. Indeed, it has been suggested that isoleucine, leucine and histidine residues could contribute to the antioxidant activity of fermented anchovy fish extracts (Najafian and Babji, 2019). A study carried out by Wu et al. on the QMDDQ peptide, from a shrimp protein hydrolysate, showed that the antioxidant potency could be related to the high number of active hydrogen sites (Wu et al., 2019). Peptide antioxidant properties are usually expressed as free radical scavenging, metal ion chelation activity and inhibition of lipid peroxidation (Jiang et al., 2020). For example, Zhang et al. showed that the VYLPR peptide has a protective effect on H_2_O_2_-induced cell damage (HEK-293 cells) (Zhang et al., 2019). Moreover, Liang et al. investigated antioxidant peptides deriving from a protein hydrolysate of *Moringa oleifera* seeds and demonstrated their protective effects on Chang liver cells exposed to H_2_O_2_ oxidative damage (Liang et al., 2020). Jiang et al. identified four peptides AYI(L) and DREI(L) from Jiuzao protein hydrolysates able to decrease ROS production in HepG2 cells (Jiang et al., 2020).

### AMPs in Intestine Infection and Inflammation

The bacterial microflora is essential for human health and the development of the mucosal immune system. In the small intestine, Paneth cells secrete α-defensins in response to bacterial antigens including LPS and muramyl dipeptide (Ayabe et al., 2000). Petnicki-Ocweija et al. showed that the bactericidal activity of crypt secretions of the terminal ileum was compromised by NOD2 gene deletion (Petnicki-Ocwieja et al., 2009). The human NOD2 protein is a cytoplasmic receptor for bacterial molecules principally expressed in Paneth cells (Lala et al., 2003) and it was identified as a susceptibility gene for Crohn’s disease (Hugot et al., 2001). Deficient expression of Paneth cell α-defensins (HD5 and HD6) may contribute to the pathophysiology of Crohn’s disease (Bevins, 2006). It has been demonstrated that mice lacking NOD2, fail to express cryptidins, equivalents of human α-defensins (Kobayashi et al., 2005). Moreover, human α-defensin expression is reduced in Crohn’s disease patients, particularly in those with NOD2 mutations (Wehkamp et al., 2005).

hBD1 was the first defensin identified in the human large intestine and in the not-inflamed colon. It was observed a reduction of hBD1 expression in inflamed mucosa in patients with inflammatory bowel diseases (Wehkamp et al., 2003). hBD1, hBD2, hBD3 and hBD4 expression has been demonstrated to be upregulated in colonic enterocytes in patients with ulcerative colitis (Fahlgren et al., 2004).

Moreover, a lot of interest has been given to the role of AMPs in the stomach, which is easily colonized by *Helicobacter pylori*. Infection by this bacterium leads to the induction of hBD2 (Wehkamp et al., 2003). It has been demonstrated that gastric epithelial cells are induced by *Helicobacter pylori* to upregulate hBD2 production (Grubman et al., 2010).

These observations make defensins very attractive from a pharmacological point of view and can offer a good starting point for future AMP clinical applications.

## Pharmacokinetic and Pharmacodynamic (PK/PD) Approach in the Evaluation of AMP Clinical Applications

### PK/PD Approach to Determine AMP Antibacterial Efficacy

PK and PD principles that determine response to antimicrobial AMPs can provide clinicians with useful information on the correct dose regimens.

Dosler and colleagues have investigated the *in vitro* activities of AMPs (indolicidin, cecropin [1–7]-melittin A [2–9] amide [CAMA], and nisin), alone and in combination with antibiotics (daptomycin, linezolid, teicoplanin, ciprofloxacin, and azithromycin) against standard and clinical MRSA biofilms, showing that AMPs improve the *in vitro* PK efficacy of traditional antibiotics (Dosler and Mataraci, 2013).

Schmidt and colleagues showed that AMPs (Onc72 and Onc112) reach several organs within 10 min after intravenous and intraperitoneal administration and the PK experiments explain the high *in vivo* efficacies of AMPs indicating their potential use for the treatment of urinary tract infections (Schmidt et al., 2016). However, these data are not sufficient to predict the exact relationship between dose, exposure, and response and translational PK/PD modeling and simulation are used to identify the most suitable dosing regimen in patients. PK/PD modeling can provide useful clues concerning the multifaceted correlation between the selected kind of AMP, the bacterium characteristics, and the reaction of the host organism. Furthermore, complicating factors can also be incorporated into the *in silico* approach thus allowing to carefully predict the right balance between bacterial killing, adverse effects, and appearance of resistance. This practice may, therefore, help to identify and to optimize the dose for novel and established antibacterial agents (Rathi et al., 2016). As previously mentioned, AMPs affect growing bacterial populations differently from antibiotics (ampicillin, ciprofloxacin, gentamicin, kanamycin, neomycin, rifabutin, spectinomycin, and tetracycline), particularly from a PD point of view (Yu et al., 2016). Moreover, Yu and colleagues, analyzing the resistance evolution by predictive model, found that differences in PD and in the mutagenic properties between AMPs and antibiotics produce a much lower probability that resistance will evolve against AMPs (Yu et al., 2018). More experiments with a variety of AMPs are needed to determine if PK/PD characteristics of AMPs can be generalized and if these characteristics are significantly different from antibiotics. However, all the available data suggest that AMPs are significantly different from antibiotics in terms of PD and mutagenic properties and are good candidates for slowing the evolution of resistance.

### PK/PD Approach to Determine AMP Efficacy in Non-Bacterial Disease

The “right” use of AMPs is imperative, not only in treating bacterial disease but also in other diseases to avoid toxicity and to limit the development of resistance. Few studies have analyzed AMP PK/PD properties in relation to no-bacterial disease. AGPSIVH, FLLPH, and LLCVAV antioxidant peptides were obtained from duck breast protein hydrolysates by Li et al. and beside the nontoxic effects exhibited digestive resistance (Li et al., 2020). Xu and colleagues used *in vitro* and *in vivo* models to study the absorption and potential antioxidant activity and the *in vivo* metabolism, respectively, of WDHHAPQLR derived from rapeseed protein (Xu et al., 2018). Koeninger and colleagues showed that hBD2 displays a good tolerability and rapidly enters the bloodstream in a model of experimental colitis after its subcutaneous administration. Thus, besides being well tolerated *in vivo*, it might not only act locally but could also have systemic effects (Koeninger et al., 2020). Several other bioactive peptides have been discovered in recent years, but their PK/PD properties are still unknown. It is therefore necessary to increase the studies to determine the PK/PD efficacy of AMPs also in non-bacterial disease.

## Drug Development and Formulation Approaches for AMP Applications

### Production and Costs - Pilot Study *vs.* Small Industrial Scale

The development of AMPs as APIs (Active pharmaceutical ingredients) has been greatly limited by their high manufacturing costs. Although the chemical synthesis of peptides has high efficiency, it is also complex and expensive. Hence, advanced natural approaches should be considered with the aim to increase the production of alternative molecules. Genetic engineering can be considered one of the most important strategies to obtain higher yields or higher quality of AMPs.

To obtain AMPs, biotechnological approaches involving competent bacteria and yeasts, as well as transgenic plants or animals, should be considered (Sinha and Shukla, 2018). Gaglione and co-workers focused on how to optimize the bacterial culturing using a new composition of culture broth. They basically considered inexpensive as well as readily available components containing well-defined amounts of each nutrient. They also substituted IPTG (isopropyl β- d-1-thiogalactopyranoside) with cheaper and more harmless sugars, such as lactose. Indeed, IPTG use might result in high-cost accumulation for industrial purposes. Altogether, the optimized bacterial culture strategy can contribute to further development to enhance the manufacturing scalability of AMPs (Gaglione et al., 2019).

However, although bacteria can produce some cyclic peptides, they do not produce disulfide-rich peptides, so that recombinant expression of cyclic peptides might be best performed in yeast- or plant-based recombinant expression systems (Thorstholm and Craik, 2012; Moridi et al., 2020).

The manufacturing cost of AMPs is estimated to be around $50‐400 per gram of amino acid produced by SPPS (Solid Phase Peptide Synthesis), thus biotechnological engineering or fermentation should give cheaper alternatives. Moreover, the identification, characterization and production of new AMPs also with biotechnology improvement is expensive from many points of view, therefore, it could be useful to perform preliminary *in vitro* screening, to evaluate physio-chemical characteristics, putative modifications in the secondary structure and putative antimicrobial activity (Moretta et al., 2020).

About the peptide drug market in 2018, more than 50 peptide drugs have been commercialized. The annual sales of peptide drugs, including the AMPs, is around 25 billion USD (Koo and Seo, 2019).

### AMP Dosage Forms

Compared to the possible sequence modifications to enhance the molecular stability, the drug delivery platform development has reported a minor attention so far. As described in literature, the dosage forms in ongoing clinical trials encompass topical gel and hydrogel, topical cream, polyvinyl alcohol-based solution for administration in the wound bed, hyaluronic acid-based hydrogel for the administration at the surgical site, oral solutions, and mouth rinse (Mahlapuu et al., 2016).

Concerning dermal administration, burn and chronic wounds can exhibit difficult control, especially in the case of upsurges caused by ESKAPE pathogens (*Enterococcus faecium*, *S. aureus*, *K. pneumoniae*, *A. baumannii*, *P. aeruginosa*, and *Enterobacter* spp). Topical administration of antimicrobials onto the skin provides many advantages since it offers a high local load of the antimicrobial. Moreover, due to the pleiotropic mechanisms of action, AMPs can contribute to fight ESKAPE infections as well as to regulate various mechanisms including the host processes of inflammation and wound healing (Kang et al., 2014; Vassallo et al., 2020). However, AMPs intended to treat chronic skin and soft tissue infections should not (i) be absorbed from the wound or infection site into the systemic circulation; (ii) rouse allergic sensitization. Topical administrations of AMPs have demonstrated to be not free of systemic side effects since the drug transport may also occur *via* skin layers and through hair follicles. Besides, the stability enhancement against enzymatic degradation needs to be assessed when peptides are developed for clinical purposes. Moreover, the membrane border of the epithelial cells includes several peptidases to be considered (e.g., leukocyte elastase, cathepsins B and D, zinc-dependent endopeptidases, interstitial collagenase), since they are characterized by a broad specificity to degrade exogen peptides (Vlieghe et al., 2010; Lam et al., 2018; Pfalzgraff et al., 2018).

### Delivery System

In the context of Drug Delivery System (DDS), peptides are playing an important role as APIs vehicles, due to the intrinsic biodegradability and biocompatibility (Giri et al., 2021). Novel DDS can also help (i) to reduce adverse side-effects, and (ii) to obtain a controlled release of the AMP (Nordström and Malmsten, 2017; Martin-Serrano et al., 2019).

#### Hydrogels – Overview and Platform Development for the AMP Dermal and Subdermal Delivery

Hydrogels (HGs) comprise materials constituted by hydrophilic as well as polymeric vehicles to entangle large amounts of water within their three-dimensional (3D) networks (Liu and Hsu, 2018). As reported in the Eur. Pharm 8^th^, gels consist of gelled liquids with suitable gelling agents. Specifically, HGs (i.e., hydrophilic gels) consist of water, glycerol, or propylene glycol-based preparations. These compounds are gelled with starch, cellulose derivatives, poloxamers, carbomers, and magnesium-aluminum silicates (European Pharmacopeia, 2016). HGs exhibit improved bioavailability for applications onto the impaired skin. Moreover, HG-based burn dressings (HBBD) appear appropriate as they provide a suitable wound covering. Thanks to a cooling sensation that occurs *via* convection and evaporation of the solvent from the wound, HBBD can also contribute to dissipating the heat that occurs from the concomitant inflammation (Fichman and Gazit, 2014; Goodwin et al., 2016). HGs have also been extensively studied since they exhibit different applicability potentials covering the cell culturing (Caliari and Burdick, 2016), the regenerative medicine (Catoira et al., 2019), and DDS developments.

**Figure 3:**
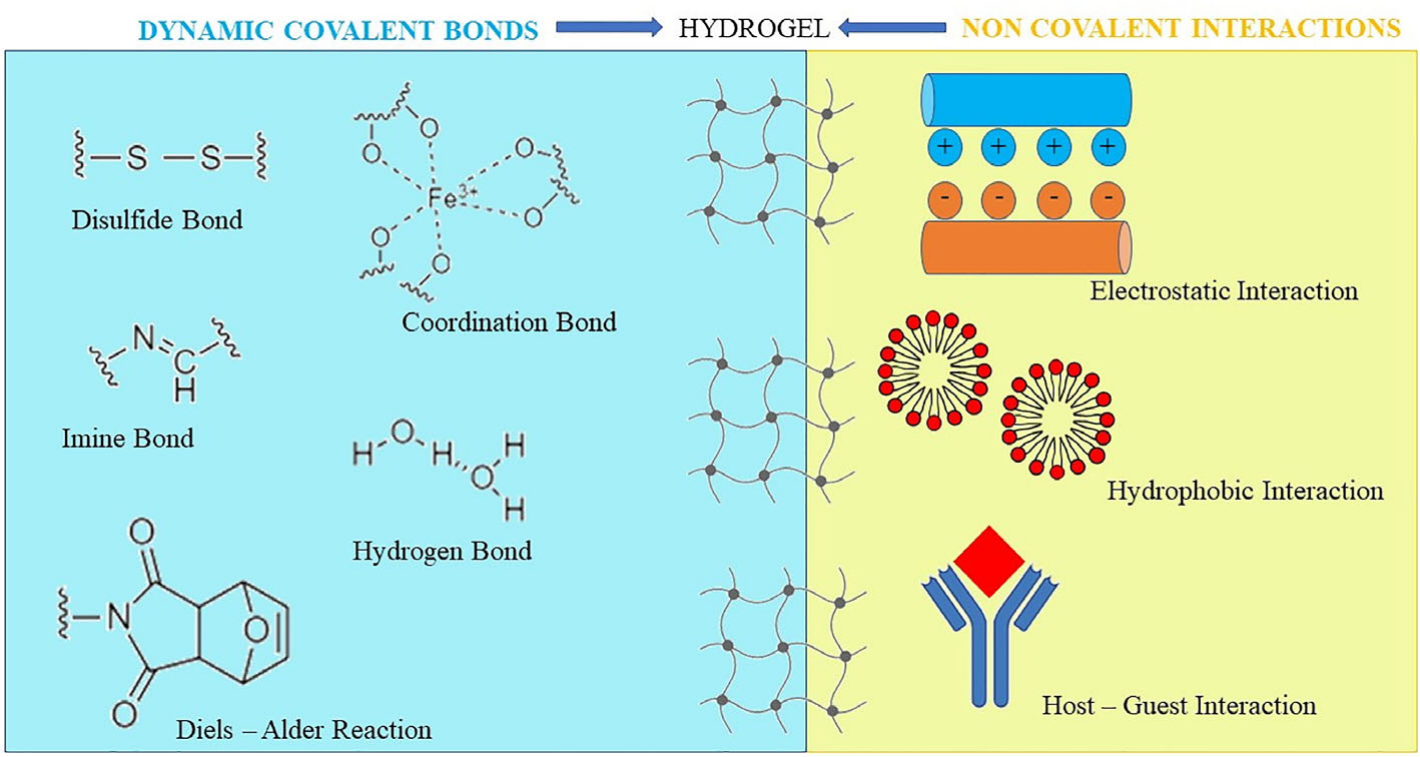
Chemical and physical bonds to obtain hydrogels. Hydrogels can also be prepared by a hybrid interaction consisting of physical interactions and/or covalent bond formation, exhibiting at the same time reversible mechanical properties and long-term stability.

After chemical interactions, such as the Michael’s addition, the Diels–Alder or Schiff base reactions, chemically-crosslinked HGs form the matrix structure (Overstreet et al., 2012) (**Figure 3**). To obtain a HG that supports the wound closure, Bian and co-workers used modified chitosan with maleic anhydride and a polyethylene glycol derivative, that was modified with benzaldehyde at both ends. *Via* a Schiff-base reaction, the obtained HG showed a shear-thinning behavior. Accordingly, it was intended to be injected/applied into/onto wounds, as it was suitable to adopt the contour as well as to seal the defects of the impaired tissue. Afterwards, the *in situ* HG solidification was promptly realized by using ultraviolet light (Bian et al., 2019).

HG can also be prepared by multiple non-covalent interactions, by which the monomeric building blocks can self-associate in ordered fibrous structures. Also, they are suitable to interact with each other forming the 3D network (Fichman and Gazit, 2014). Moreover, thanks to a self-assembly skill of polymers e.g., *via* changing pH and temperature, the physical cross-linking method favors the formation of weaker and stimuli-responsive HG. Hence, HG can temporarily modify the structure due to the solicitation of external mechanical forces and the shear-thinning behavior (Yan et al., 2010).

Since a substantial change in volume is usually not observed, HGs are also suitable as injectable vehicles (Manna et al., 2019). Moreover, HG can also polymerize *in situ* becoming a shear-thinning material after injection, allowing, therefore, AMP delivery. The *in situ* forming HG was demonstrated useful for ophthalmic applications, as well as to support the wound-healing after surgical operations (Travkova et al., 2017). The widely used materials and techniques for surgical closure purposes may contribute to providing some drawbacks. Hence, contaminations by impurities from air or from a fluid leakage can contribute to microbial infection harm (Rajabi et al., 2020). Moreover, medicated HG can release AMPs at the site of action after disruption of the inner matrix by erosion, swelling, or *via* enzyme interactions (Chen M. H. et al., 2017).

Li and co-workers formulated a thermosensitive HG constituted of biodegradable poly (l-lactic acid)-Pluronic L35-poly (l-lactic acid) for cutaneous wound-healing treatment, to investigate whether AMPs encapsulated in this HG formulation demonstrated efficient candidates in wound healing management. They used a type of multifunctional human-derived AMP (i.e., AP-57), with a broad-spectrum antimicrobial activity as well as an immune regulation ability. The AP-57 peptide was enclosed first in biocompatible nanoparticles, named AP-57-NPs. Subsequently, to facilitate their application in cutaneous wound repair, the AP-57-NPs were further encapsulated in a HG matrix (AP-57-NPs-H). As reported, the *in situ* gel-forming system exhibited *in vitro* a low cytotoxicity and a sustained drug release behavior. After applied to the wound, the formulated peptide achieved additional characteristics, such as a non-flowing gel that consequently become a sustained drug depot. Li and co-workers also demonstrated wound-dressing properties of this formulation. The effect of the formulated AMP was then investigated on full-thickness excision wound using the Sprague-Dawley^®^ male and albino rat models. At last, the obtained DDS was effective on the wound, and rat models reported a complete wound closure (Li et al., 2015).

A different method to obtain HG in the aqueous phase is the mussel-inspired polydopamine chemistry. A study of Khan and colleagues reported the use of catechol, instead of dopamine, as a cross-linker with amine-rich polymers to prepare thin films. Catechol is less expensive than dopamine; hence, it was used with ϵ-poly-L-lysine (EPL), a natural AMP produced by *Streptomyces albulus*, to fabricate HG with antimicrobial properties. EPL-catechol HG showed *in vitro* antimicrobial and antibiofilm properties against multidrug-resistant *A. baumannii* associated with a good biocompatibility with a mouse myoblast cell line and *in vivo* reduced the bacterial load and improved wound healing when topically applied on the skin of a mouse with a second-degree burn wound also infected with multidrug-resistant *A. baumannii* (Khan et al., 2019). Lee and colleagues engineered nanoparticle-HG corneal implants containing the human AMP LL-37: although *in vivo* studies have not already been carried out, this device could inhibit *in vitro* HSV-1 attack to ocular cells (Lee et al., 2014). An example of insect AMP formulated as HG was recorded from *Lucilia sericata*, in both wound bandages and cosmetics to hinder dermatological pathogens (Mylonakis et al., 2016).

#### Cubosome Delivery System

**Figure 4:**
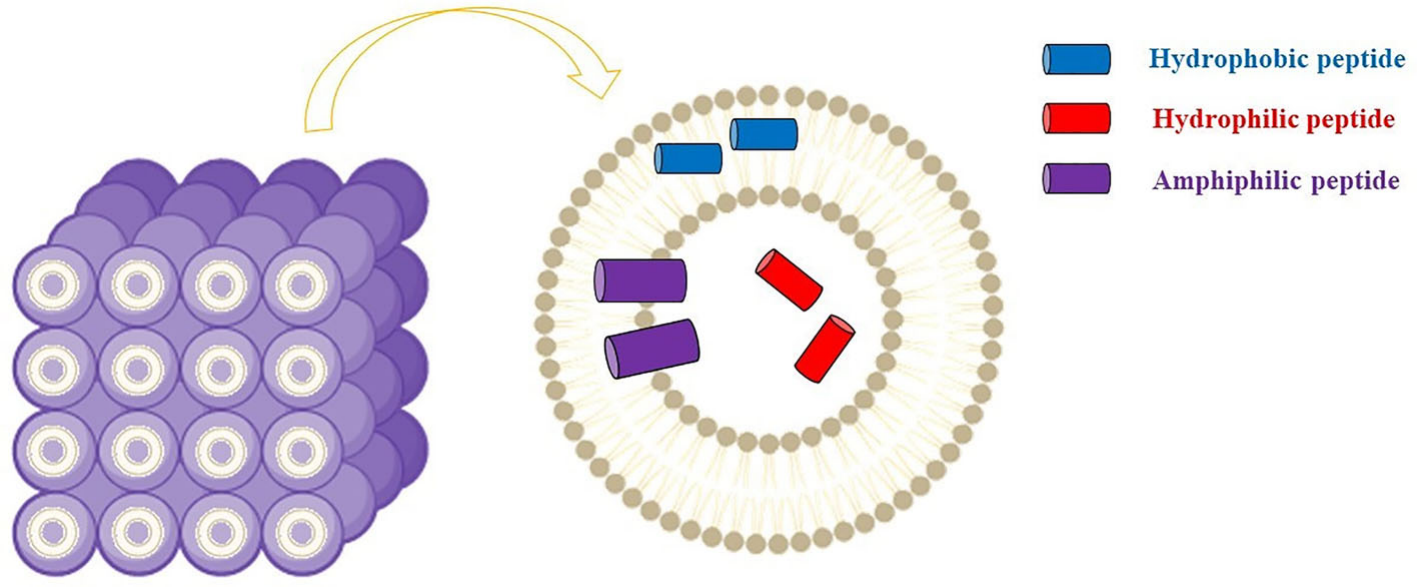
Cubosomes comprise curved lipid bilayers with a well-defined disposition and divided into two internal aqueous channels that can be exploited by antimicrobial peptides. Figure created with Biorender.com.

Cubosome represents alternative drug delivery scaffold systems consisting of a curved continuous lipid bilayer that can be realized with amphiphilic molecules. The most common amphiphilic lipid systems can comprise water and glyceryl monooleate (GMO) (2,3-Dihydroxypropyl (9Z)-9-octadecenoate) (1-Oleoyl-rac-glycerol | C_21_H_40_O_4_ | ChemSpider). Similar dispersions show several self-assembly dispositions, among which the bicontinuous cubic phases (**Figure 4**).

Practically, bicontinuous cubic phases can be obtained by dispersing the amphiphilic lipid system into the aqueous phase using e.g., ultrasonication or homogenization. Subsequently, a dispersed gel is obtained, known as cubosome (CB) (Karami and Hamidi, 2016). As a result of the hydrophobic effect, thermodynamically stable structures with a well-defined disposition of each component (i.e., the cubic liquid crystalline gel) are realized (**Figure 3**). These nanostructures have demonstrated suitable for loading hydrophilic, hydrophobic, as well as amphiphilic cargos.

More importantly, CB can include bioactive compounds, as the structure provides a significantly higher membrane surface area to loading proteins (Barriga et al., 2019).

Anatomically, the stratum corneum represents a strong barrier for the transdermal drug delivery of topically applied drugs, due to the presence of the external and highly organized skin layer. The ability of CB to adhere to the stratum corneum makes CB effectively useful in topical drug delivery for mucosal tissues (Gaballa et al., 2020). The structure and properties of CBs provide a promising vehicle for transdermal drug delivery especially for skin infections (Zeng et al., 2012; Meikle et al., 2019).

AMPs can be adsorbed onto the CB structure that usually shows a slightly negative charge. For instance, Boge and co-workers demonstrated that the GMO based-CB structure contributes to protecting the AMPs from proteolytic degradation, improving their bioavailability after topical administration. Furthermore, they found that AMPs loaded onto CB are highly released in the milieu whether *P. aeruginosa* or human neutrophil elastases are present.

The authors also reported a study investigating CB interaction with both a bacterial membrane model and *E. coli*’s membrane, to further understand how the interaction between AMPs and the membranes can be accomplished. The authors suggested that the bactericidal effect was due to physical interaction between the product and the bacterial membrane and not solely to the release of the peptide. Moreover, they noted that the presence of LL-37, the chosen AMP, constituted of a secondary structure of a linear α-helix increased the affinity of CB to bacterial membranes (Boge et al., 2017; Boge et al., 2019a; Boge et al., 2019b).

Many papers have reported that the composition of GMO-based CB generally involves the use of stabilizer molecules. The stabilizer avoids the aggregation of hydrophobic portions with the external aqueous media and consequently helps to reach a thermodynamically stable form (Gaballa et al., 2020). Pluronics, especially poloxamer 407 (F127), represent the most used stabilizing agents. This nonionic copolymer vehicle comprises a central hydrophobic chain of polypropylene oxide with a molecular weight of approximately 12.6 kDa and lateral hydrophilic chains of polyethylene glycol (Barriga et al., 2019). The clinical application of GMO-based CB stabilized by F127 may be limited due to concentration-dependent cytotoxicity. Moreover, F127 may also show hemolytic effect, as well as a poor biodegradability. A novel stabilizer-free antimicrobial nanocarrier was developed by Zabara and co-workers, by dispersing GMO in water using ultrasonication and combining the AMP LL-37 by spontaneous integration in the internal nanostructure. Comparing the new system to the GMO-based CBs stabilized with F127, they found that the stabilizer-free nanocarrier showed cytocompatibility and a higher antimicrobial effect, especially against the tested Gram-negative pathogens, among which *P. aeruginosa* CIP A22 DSMZ 25123 strain (Zabara et al., 2019).

#### Other Drug Delivery Systems

Some negative aspects are related to the lipid-based nanocarriers: beside the poor stability, they are also susceptible to aggregation *in vitro* and to esterase activity. This last aspect might also affect the relationship between the *in vitro* and the *in vivo* controlled release of the cargo. Subsequently, materials alternative to lipids have been explored including self-assembled polymeric nanocarriers for preparing both vesicular and bicontinuous systems. Compared to lipids, the block polymeric structures (BPS) can be synthesized from an expansive pool of amphiphilic monomers. Therefore, BPSs, called also polymersomes, have demonstrated high flexibility to functionalization, along with well-defined structures that can be distinguished in both hydrophobic and hydrophilic sections. Hence, the BPS can exhibit substantial rewards involving both mechanical and chemical stability (Allen et al., 2019).

**Figure 5:**
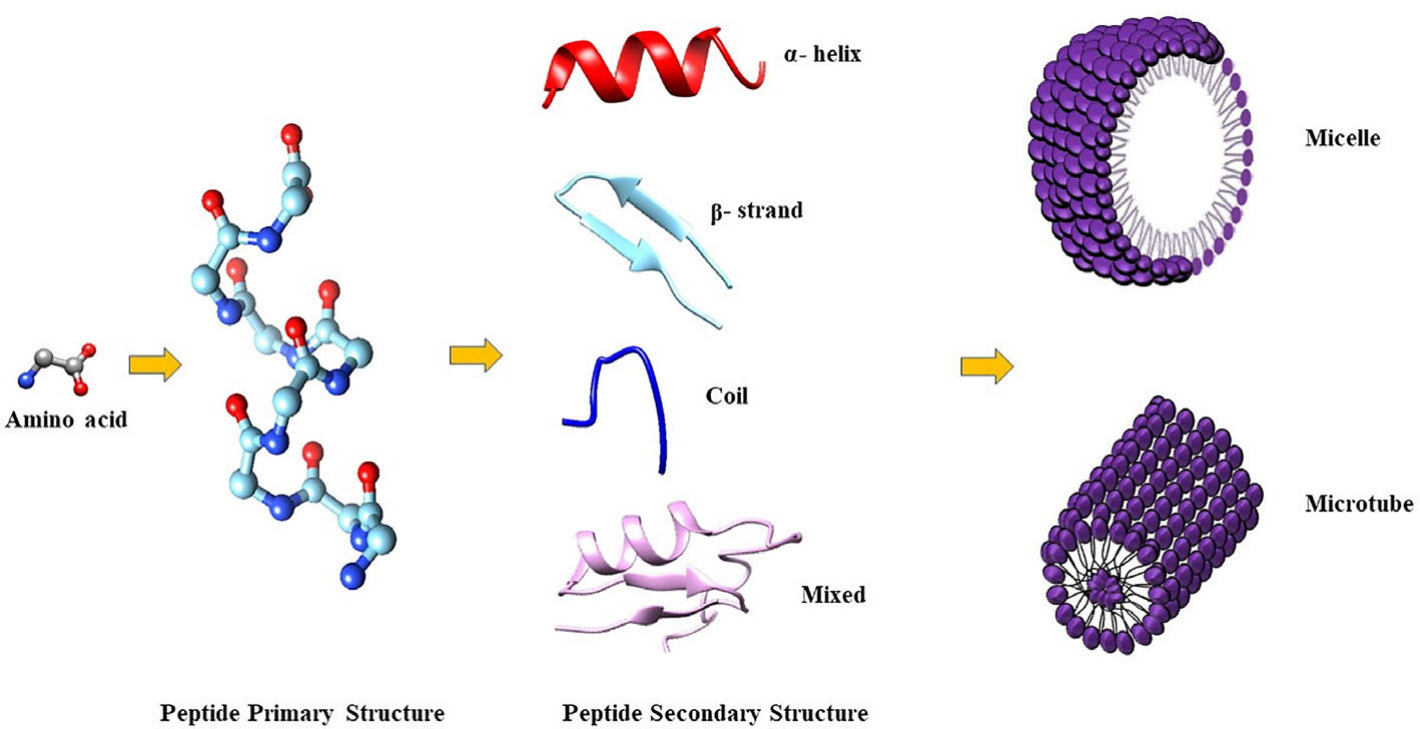
Arrangement of peptide amphiphiles in self-assembling nanostructures (e.g., micelles and microtubes), which can contain and release APIs. Adapted from Song et al. (2017). Figure created with UCSF CHIMERA software (Pettersen et al., 2004).

Most AMP formulations in ongoing clinical trials belong to semi-solid preparations for external use (Koehbach and Craik, 2019; Koo and Seo, 2019; Sheard et al., 2019). Hence, among the topical formulations, topical gel formulations are often mentioned in several research works to treat e.g., chronic skin and soft tissue infections. Moreover, proteins but also longer peptides ranging between 20 to 30 amino acids can also self-assemble naturally to achieve α-helices or β-sheets motifs. Likewise, two antiparallel β-strands can fold in β-hairpin motif, which contributes to creating higher-ordered fibers and pH-responsive active pharmaceutical ingredient vehicles. Recently, specific functional peptides have been synthesized and utilized as useful nanomaterials. Particularly, important properties have characterized a special group of synthetic peptides called peptide amphiphiles (PAs). They essentially consist of four sequences: (i) a hydrophobic tail (e.g., palmitic acid residue); an internal portion able to form β-sheets, which comprises (ii) an amino acid sequence to promote through hydrogen bond the formation of fibril-like structures; (iii) a spacer containing charged amino acids to allow solubility and cross-linking (Cui et al., 2010); at the opposite end of the structure, (iv) the hydrophilic head can be found that triggers the signaling for the biological response. Due to the molecular organization and the chemical characteristics, PAs can organize spontaneously in a nanostructure using a folding-like behavior to form specific nanostructures, including micelles and microtubes (**Figure 5**).

Hence, to stabilize the system in a lower energy state, PA molecules can organize the alkyl chains away from the aqueous environment, exposing externally the hydrophilic portion. PAs have attracted special interest as drug carriers due to their (i) advantage of a unique structure of assemblies, (ii) abundant molecular structures, and (iii) ability to give biological functions (Song et al., 2017). Additionally, the self-assembly aptitude of di-phenylalanine (Di-Phe) building-blocks can be used to obtain diverse supramolecular nanostructures, such as nanofibrils, or nanowires.

These structures have demonstrated large applicability due to their biocompatibility, high loading capacity and simplicity to obtain the self-assembled nanostructures. Furthermore, as reported by Schnaider and co-workers, nano-assemblies formed by Di-Phe exhibited an intrinsic antibacterial activity (Schnaider et al., 2017).

Such nanostructures can considerably enhance the active pharmaceutical ingredient stability since they become less sensitive towards enzymatic degradation. Likewise, most AMPs forming α-helices or β-sheets could be inserted into supramolecular nanostructures. This strategy might contribute, therefore, to a suitable delivery of AMPs without using additional vehicles and their molecular stability. Upon contact with the pathogen, the peptide nanostructure is disrupted, especially from peptidases, and releases the AMP.

Also inorganic nanomaterials (metal and metal oxide nanoparticles, silica, nanoclays, and carbon-based nanomaterials) have investigated as AMP delivery systems, because they shield the molecules from degradation and avoid peptide aggregation or conformational changes that could inactivate them (Nordström and Malmsten, 2017). Furthermore, they have the ability to control the drug release (thanks to well-defined pore sizes and forms (Vivero-Escoto et al., 2010)) increasing bioavailability and reducing toxicity (Nordström and Malmsten, 2017). In addition, several nanoparticles have been shown to have antimicrobial properties against both Gram-negative and -positive bacteria, suggesting that the complex AMP-nanoparticle may have a synergistic impact (Hajipour et al., 2012). Another synergistic effect could be achieved by a close interaction between AMP and antibiotics, which can be carried together in mesoporous silica nanoparticles with good chemical stability and biocompatibility, even though it is always important to consider the chemical nature of these nanoparticles, the dosage, and the administration route (Nordström and Malmsten, 2017).

## Administration Routes

Compared to other routes of administration, the intramuscular, or the subcutaneous routes may not require too much stability of the peptide. Indeed, AMP physicochemical and biological characteristics could be taken into less account in these routes of administration, while size, permeation through gastrointestinal membrane, poor stability to gastric pH and susceptibility to proteolytic enzymes make the oral administration much difficult (Schiffter, 2011).

Hence, injection represents the best route of administration for most of the AMPs (Di, 2015). However, the intravenous administration certainly exposes the peptides to the esterase and peptidase activity present in serum (Vlieghe et al., 2010; Fosgerau and Hoffmann, 2015).

The oral route remains a patient-friendly option, due to the non-invasive and painless administration. However, considering few exceptions, the oral pharmaceutical technologies have not shown radical improvements regarding the AMP formulation to increase their bioavailability. The principal efforts concern the peptide stability due to the presence of pancreatic peptidases, e.g. α-chymotrypsin, trypsin and pancreatic elastase secreted from the pancreas into the gastrointestinal tract (Vlieghe et al., 2010; Aguirre et al., 2016; Malhaire et al., 2016).

Furthermore, high dosage and low systemic exposure allow minimizing systemic side effects when a drug is formulated for the lung administration. Inhaled medications of peptides have demonstrated superior in terms of rapid onset (Larijani et al., 2005). Peptide macrocycles with antimicrobial effect working as protein epitope mimetics can also be formulated for inhalation, due to appropriate chemical stability. The POL6014, a neutrophil elastase inhibitor (i.e., Murepavadin^®^), can be administered *via* eFlow^®^ nebulizer system to treat cystic fibrosis lung infections and it is currently in Phases I/II (NCT03748199, 2018).

In conclusion, as reported above, topical applications involving AMPs loading in nanoparticles, hydrogels, creams, gels and ointments represent the most used and best developed AMP applications and further studies are needed to exploit new suitable administration routes.

## AMPs in Ongoing Clinical Trials

We have described several AMPs approved for clinical applications. However, many others, both natural and synthetic, are still under clinical trials (**Table 3**). Preliminary results suggested that many AMPs could be useful alone or in synergy with common antibiotics to prevent or treat several diseases, but most of the studies are still ongoing or were stopped because of issues that can be solved, including unfavorable pharmacokinetic profile or unexpected side effects (Browne et al., 2020; Dijksteel et al., 2021).

**Table 3:** List of the AMPs in ongoing clinical trials.

AMP	Peptide structural characteristic	Ongoing Clinical Trials
Bacitracin	Natural cyclic peptide	Phase IV
Pexiganan	Natural linear peptide	Phase III
Omiganan	Indolicidin derivative peptide	Phase III
LL-37	Natural α-helical peptide	Phase II
LTX-109	Synthetic Antimicrobial Peptidomimetic	Phase II
Brilacidin	Synthetic peptide	Phase II

Below we reported some recent clinical trials, focusing the attention on studies still in progress.

Bacitracin, a natural cyclic AMP from *Bacillus subtilis*, is currently reported in several ongoing studies of phase IV to treat Gram-positive bacterial infections (Bacitracin - ClinicalTrials.gov). These studies are evaluating bacitracin (i) in subjects with minor, second-degree burns, for topical use and in combination with a second ointment of collagenase; (ii) as an ointment to treat skin infections in combination with medical-grade honey; (iii) as an ointment for topical antibiotic therapy after eyelid surgery and to evaluate the use of antibiotic prophylaxis in presence of antibiotic side effects and antibiotic allergy; (iv) as topical antibiotic irrigation to reduce surgical site infections and in combination with neomycin and polymyxin (Neomycin^®^) for postoperative urinary tract infections and to extend the antimicrobial effect to Gram-negative bacteria; (viii) to evaluate the efficacy of preoperative oral antibiotic prophylaxis for preventing surgical site infections in elective colorectal surgery (combination of Bacitracin and the antibiotic Neomycin). Another clinical trial was ongoing to evaluate the topical use of bacitracin to reduce surgical site infections in midfacial fracture surgery, but in April 2020 this trial was closed because of bacitracin toxicity. Other phase IV studies involving bacitracin are aimed to the treatment of (v) facial burns, (vi) in combination with topical tranexamic acid (i.e., 5%, and 25%), and (vii) with polymyxin B (Polysporin^®^) to evaluate the use of Biofine^®^ cream on wounds due to cryotherapy for removing actinic keratosis lesions (Bacitracin - ClinicalTrials.gov).

Pexiganan, a linear AMP, is under investigation in four phase III studies for the treatment of diabetic foot ulcers using topical cream formulations (Gottler and Ramamoorthy, 2009).

Omiganan, an indolicidin derivative (Sader et al., 2004), has been tested in a total of sixteen studies and thirteen of them have been completed. Looking at the completed ones, three phase III studies have been reported, among which two were aimed to evaluate the efficacy of AMPs as topical gel formulation to treat rosacea. The third phase III study concerned the treatment of catheter colonization, and prevention of bloodstream infections if applied to the skin surrounding the insertion.

The innate immunity of mammals comprises also the cathelicidins as a distinct class of proteins. Like defensins, although their structural features clearly distinguish them from defensins, cathelicidins act as precursor molecules that can release an AMP after proteolytic cleavage (Dürr et al., 2006).

The human cathelicidin-derived AMP, named LL-37, belongs to the class of α-helical AMPs. Currently it can be found on a Phase II clinical trial by Promore Pharma (Promore Pharma AB, Sweden) evaluating LL-37 safety and tolerability in patients with venous leg ulcers (Grönberg et al., 2014; Sierra et al., 2017; Koo and Seo, 2019). It is also under investigation in patients with diabetic foot ulcers (LL-37 - ClinicalTrials.gov).

The synthetic AMP LTX-109 represents a novel class of very short AMPs. It has been described as a synthetic antimicrobial peptidomimetic and has entered the phase II clinical studies (Isaksson et al., 2011) with the aims (i) to assess the clinical and microbiological response of two LTX-109 dosages (i.e., 1%, and 2%) formulated as a topical gel (Lytixar™) for the treatment of non-bullous impetigo; (ii) to evaluate the safety, local tolerability, and efficacy of 1%, 2% and 3% LTX-109 gel formulations for the anterior nare delivery in patients who are carriers of MRSA/MSSA (methicillin-susceptible *S. aureus*); (iii) defining the magnitude of systemic absorption when LTX -109 is applied to the anterior nares as a topical gel; (iv) to evaluate the safety and tolerability of topical Lytixar™ formulation onto uncomplicated skin infections, as well as to investigate both the clinical and microbiological effect of Lytixar™ in patients with uncomplicated skin infection by Gram-positive and to determine the degree of systemic absorption of LTX-109. A further trial in recruiting phase is aimed to demonstrate the safety of a percutaneous application of a 3% gel cream of LTX-109 in *Hidradenitis suppurativa*, to identify the clinical responses and the influences of specific parameters, including age, disease duration, and body mass index (LTX-109 - ClinicalTrials.gov).

Brilacidin is a synthetic AMP, successfully tested in Phase II clinical trials for treatment of acute bacterial skin and skin structure infections. A recent work demonstrated that Brilacidin displays an antiviral activity, inhibiting SARS-CoV2 virus in Vero African green monkey kidney cells and Calu-3 human lung epithelial cells and showing a synergistic inhibitory activity in combination with the antiviral Remdesivir (Bakovic et al., 2021). A Phase II clinical trial is going to start to assess the efficacy and safety of Brilacidin on patients with moderate or severe SARS-CoV-2 infection, hospitalized with respiratory difficulty but not requiring high-level respiratory support (Brilacidin - ClinicalTrials.gov).

## Conclusions

AMPs can be considered unconventional therapeutic small molecules which have attracted great interest in recent years because of their promising potential, as they can be used as alternative or complement approaches for treatment of microbial infections. Due to their potency, broad-spectrum activity, different sources available in nature, lack of rapid development of resistance, low accumulation in tissue and rapid killing activity, these peptides show several advantages over conventionally used antibiotics. Moreover, AMPs also display immunomodulatory, antioxidant and anti-inflammatory activities and, for this reason, researchers are devoting considerable efforts to implement the use of AMPs as commercially available drugs. This review examined the features of AMPs, their mechanisms of action and their sources, highlighting their antimicrobial activity against several pathogens involved in human infections. Thus, the efficacy and potentially applicability of AMPs in human diseases has been analyzed. Particularly, we examined the beneficial role of several AMPs in the treatment of skin infections, but we also reviewed their potential use in respiratory diseases and oxidative-stress disorders, such as obesity, diabetes and chronic inflammatory intestinal disorders. Indeed, AMPs display several potential applications in medicine, since they can regulate pro-inflammatory reactions, stimulate cell proliferation, promote wound healing by modulating the cell migration, angiogenesis, chemotaxis and cytokine release. On these bases, pharmaceutical companies are performing great efforts to develop AMPs as therapeutic agents, improving their chemical and metabolic stability, setting up smart and novel formulation strategies, with the aim to improve AMP delivery and, consequently, their activity.

## Author Contributions

Conceptualization: PF. Writing - original draft preparation: PF, AMP, AP, CS, RS, MDM. Writing - review and editing: AMP, AP, CS, RS, MDM, AF, DL, AV, HV, AS, PF. Supervision: PF. All authors contributed to the article and approved the submitted version.

## Funding

This research was supported by the Italian Ministry of Instruction, University and Research (MIUR) within the frameworks of three projects (PON R&I 2014-2020, protocol ARS01_00597; PRIN 2017, protocol Prot. 2017AHTCK7; PO FESR BASILICATA 2014-2020 “AAA: SAFE SOS” D.D. 12AF.2020/D.01255-9/11/2020).

## Conflict of Interest

CS, RS, AF and PF were employed by Spinoff XFlies s.r.l.

The remaining authors declare that the research was conducted in the absence of any commercial or financial relationships that could be construed as a potential conflict of interest.

## Abbreviations

ACE, Angiotensin-Converting Enzyme I; AMP, Antimicrobial Peptide; APD, Antimicrobial Peptide Database; API, Active pharmaceutical ingredient; DDS, Drug Delivery System; Di-Phe, di-phenylalanine; EGFR, Epidermal Growth Factor Receptor; EPL, ϵ-poly-L-lysine; GMO, Glyceryl Monooleate; HBBD, HG-Based Burn Dressings; hBD, Human β defensin; HG, Hydrogel; IPTG, Isopropyl β- D-1-Thiogalactopyranoside; LPS, Lipopolysaccharides; MRSA, Methicillin-resistant Staphylococcus aureus; PD, Pharmacodynamics; PK, Pharmacokinetics; SARS-CoV-2, Severe Acute Respiratory Syndrome Coronavirus 2; SCID, Severe Combined Immunodeficiency; TLR, Toll-like receptor; WHO, World Health Organization; BPS, block polymeric structure; CB, cubosome; CMC, Critical Micelle Concentration; PA, peptide amphiphiles; SPPS, Solid Phase Peptide Synthesis.
